# Nanostructures as Radionuclide Carriers in Auger Electron Therapy

**DOI:** 10.3390/ma15031143

**Published:** 2022-02-01

**Authors:** Nasrin Abbasi Gharibkandi, Joanna Gierałtowska, Kamil Wawrowicz, Aleksander Bilewicz

**Affiliations:** Institute of Nuclear Chemistry and Technology, 03-195 Warsaw, Poland; n.abbasi@ichtj.waw.pl (N.A.G.); j.gieraltowska@ichtj.waw.pl (J.G.)

**Keywords:** Auger electron therapy, nanostructures, radionuclides, organic nanocarriers, inorganic nanocarriers

## Abstract

The concept of nanoparticle-mediated radionuclide delivery in the cancer treatment has been widely discussed in the past decade. In particular, the use of inorganic and organic nanostructures in the development of radiopharmaceuticals enables the delivery of medically important radioisotopes for radionuclide therapy. In this review, we present the development of nanostructures for cancer therapy with Auger electron radionuclides. Following that, different types of nanoconstructs that can be used as carriers for Auger electron emitters, design principles, nanoparticle materials, and target vectors that overcame the main difficulties are described. In addition, systems in which high-Z element nanoparticles are used as radionuclide carriers, causing the emission of photoelectrons from the nanoparticle surface, are presented. Finally, future research opportunities in the field are discussed as well as issues that must be addressed before nanoparticle-based Auger electron radionuclide therapy can be transferred to clinical use.

## 1. Introduction

Cancer has been one of the main causes of death worldwide during the past decade. Surgery, external radiation therapy, and chemotherapy are still the major and first-line treatments in oncology used for malignant tumors. In the case of aggressive and proliferative cancers, in which metastatic sites are frequently spread throughout the whole body, chemotherapy is usually the main and only applicable therapy. Its efficacy may be limited by the tumor type, administered dose, overall health, and severe side effects. Unfortunately, the low tumor specificity of cytotoxic drugs also causes damage to surrounding healthy cells. Moreover, different types of cancer cells exhibit acquired or innate resistance to administered drugs. Therefore, it is necessary to search for new, less toxic, and more effective drug therapies designed to kill tumor cells specifically. Current studies are focused on the development of new approaches to treat cancer cells selectively, without affecting the healthy tissues. These methods also include targeted radionuclide therapy (TRT) in which cancer cells are killed by corpuscular radiation (electrons or α particles) emitted by radionuclides conjugated to biological vectors, such as peptides, monoclonal antibodies, their fragments, or other small biologically active molecules. Effective targeting with biological vectors can be achieved due to their ability of recognition and subsequent binding to transmembrane proteins, which are expressed (or overexpressed) in cancer cells and frequently play a crucial role in tumor physiology and pathomorphology. Typically, α or β particles emitting isotopes are used in TRT. In the case of solid tumors, targeting agents labeled with radionuclides are concentrated within the tumor, while a small dose of radiation is deposited into surrounding normal tissues.

It is widely accepted that metastasis is one of the leading reasons for recurrence and consequent cancer mortality [1]. It may contain a small number of cells and be spread throughout the organism, even over long distances by lymphatic or blood vessel penetration. Unfortunately, the tissue range of β^-^ particles is about several hundred cells in length and thus, it is not the most suitable for the treatment of single cells or even small clusters of cells. Furthermore, non-specific irradiation of hematopoietic stem cells in the bone marrow limits the dose of radioactivity that can be safely administered to the patients, influencing the low effectiveness of TRT and consequently precluding the use of β^-^ emitters in this case. Treatment of small tumors, clusters of cells, or micrometastases can be more effective with α particles. The potential use of α-emitting radionuclides offers several significant advantages. The tissue range of α particles is only several cells in diameter (40–100 µm), which in combination with their high linear energy transfer (LET = 100 keV/µm), results in very high radiocytotoxicity. Their high radiobiological effectiveness is due to causing lethal double DNA (deoxyribonucleic acid) strand breaks. In contrast to β^-^ radiation, α particles possess high cytotoxic effectiveness even in hypoxic regions within a tumor [2]. Despite achieving notable therapeutic effects such as prostate cancer metastases treatment with ^225^Ac-PSMA-617 (prostate specific membrane antigen) radiobioconjugate [3], this method cannot be used more widely due to the low availability of α-emitters. Current supplies of the most popular in nuclear medicine α-emitter (^225^Ac) remain limited to isolated by-products from nuclear weapons processing and power plant development within the USA and Russia. The actual production level of ^225^Ac (~1.7 Ci per year) is only adequate for preclinical studies and for a limited number of clinical trials [4]. Recent intensive work on cyclotron production of ^225^Ac in the spallation reaction of ^232^Th and by proton irradiation of ^226^Ra target did not yield the expected results, mainly due to the contamination of ^225^Ac by long-lived ^227^Ac (t_1/2_ = 20 y).

Auger emitters can overcome all of the current issues related to α emitters while still providing a similarly high LET component for therapeutic application. It was found that the Auger electron emitters located in the DNA strand were more radiotoxic than α particle emitter ^210^Po [5]. Due to their very short range, the Auger electrons have properties similar to particles exhibiting high LET. As a result, they can induce several double-stranded DNA breaks over the distance of several nucleotides [6]. Furthermore, in contrast to α and β^-^ radiation, the Auger radiation emitters maintain low toxicity when transported in the blood or bone marrow, but become extremely efficient when incorporated into the DNA of the target cells. This type of targeted radionuclide therapy with Auger-electron emitters is known as Auger therapy.

**Figure 1:**
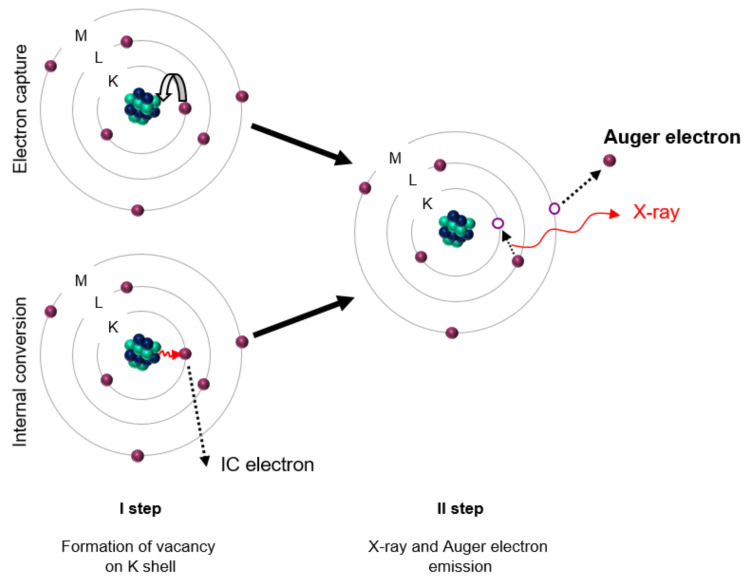
Emission of Auger and conversion electrons during electron capture and internal conversion. In the first step, the vacancy formation on the K shell by internal conversion or electron capture processes leads to atom excitation. In the following step, the atomic relaxation to the ground state occurs via radiative (X-ray) and nonradiative processes (i.e., Auger electron emission). Adapted from [7].

The sources of Auger electrons are radionuclides, which decay through the electron capture or internal conversion processes, leaving vacancies in electron shells that are rapidly filled by electrons from external orbitals. These electron transitions are accompanied by the characteristic X-rays or cascades of Auger electrons. The mechanism of this phenomenon is shown in Figure 1.

Typically, an average of 5 to more than 35 Auger electrons—with energies between a few eV to around 1 keV—are emitted per one decaying atom. Linear energy transfer of Auger electrons ranges from 4 to 26 keV/µm [7,8], and therefore, Auger electrons are similar to α particles and induce significant damaging effects in cells. Since the path length of Auger electrons is relatively short (compared to the size of the cell), minimal toxicity to surrounding non-targeted cells is observed. Moreover, after emission of Auger electrons, the daughter nuclide is in the form of an extremely reactive, highly positively charged cation, such as +35 in the case of ^195^Pt^35+^ after the decay of ^195m^Pt. Neutralization of this cation is achieved by the electrons from water in the cytosol. Subsequently, secondary radicals are formed, and the enhancement of therapeutic efficacy can be observed. Auger emitters are therefore promising candidates for targeted radionuclide therapy. Indeed, it is possible to inject about 10-fold greater radioactivity of Auger emitters than β^-^ particle emitters without toxic side effects. As a result, radiopharmaceuticals labeled with Auger emitters are expected to become widely used in radionuclide therapy in the near future. The basis for this assumption includes their high cytotoxicity and therapeutic efficacies. Another advantage is the reported sufficient availability of many low-energy electron-emitting radionuclides in a non-carrier-added form, with variable physical half-lives and known chemical properties. Furthermore, radiopharmaceuticals labeled with Auger emitters have very low autoradiolysis levels (even at high specific activity), which is a substantial benefit [8].

Although the potential for cancer therapy with Auger electrons has been widely established, there have been many barriers to the successful introduction of this therapy. Because most of the energy released by Auger electrons is deposited closely to the decay site [9], the successful use of Auger electron emitters in the therapy requires their precise delivery not only into the desired target cell but even more specifically to the sensitive organelles inside the cells. DNA in the cell nucleus is usually considered to be the most sensitive target for the delivery of Auger electron emitters. The double-strand DNA helix has a diameter of 2 nm and in a typical Auger radiation decay, the highest energy deposition occurs within spheres of 1–2 nm [10]. Hence, the calculated local energy deposition of an Auger emitter incorporated into DNA would hit both strands with an energy of 1.6 MGy or higher. For ^125^I decays associated with DNA, this translates into “one decay = one double-strand break” [11]. Similar to the α radiation path through the cell nucleus, genetic information is lost in these double-strand breaks due to the destruction of several nucleotides in both DNA strands [12]. As mentioned, it is crucial that Auger radiation, which has high α-like cytotoxicity when located close to DNA, has low toxicity outside the cell nucleus [13].

Several radionuclides emitting Auger electrons are available for the radiolabeling of biological vectors (Table 1). The energy of emitted electrons, the half-life of the radionuclide, the ratio of emitted photons (penetrating forms of radiation) to electrons (non-penetrating), the possibility of easy binding to the biomolecule and the ease of producing high activity of the radionuclide in the non-carrier added form are the parameters that should be considered when selecting the appropriate radionuclide for Auger electron therapy.

The listed radionuclides can be easily attached to biomolecules by halogenation reactions (^77^Br, ^123,124,125^I) or by chelation with DTPA (diethylenetriaminepentaacetic acid), DOTA (2,2′,2″,2‴-(1,4,7,10-Tetraazacyclododecane-1,4,7,10-tetrayl)tetraacetic acid), NOTA (2,2′,2″-(1,4,7-triazacyclononane-1,4,7-triyl)triacetic acid), or sulfur ligands. However, in the case of labelled monoclonal antibodies, the obtained specific activity is too low for therapeutic application. For example, the specific activity of ^111^In-labeled trastuzumab does not exceed 0.24 MBq/μg, resulting in only 1 of 50 molecules being radiolabeled [14]. As a result, a high proportion of HER-2 (human epidermal growth factor receptor 2) receptors were bound by non-radiolabeled immunoconjugates, limiting the cytotoxic efficacy. On the other hand, increasing the number of chelators conjugated with trastuzumab to increase specific activity may not be beneficial due to a possible decrease in antibody immunoreactivity [15]. Another approach to increase specific activity and optimize the efficacy of Auger electron targeted radionuclide therapy is to develop nanostructure-based delivery systems with many chelators attached to polymers, dendrimers, or inorganic nanoparticles working as carrier molecules. 

Nanomedicine is one of the fastest growing scientific fields in the design of new diagnostic and therapeutic methods. Anticancer drug delivery systems represent a widely investigated research area in the context of nanoparticles medical application. The surface of nanoparticles can be modified with proper vectors (such as antibodies, peptides, or small biologically active molecules) which have high affinity to the receptors on tumor cells, and the number of these targeting ligands bound to one nanoparticle can be much more than one [16]. Specific binding to transmembrane receptors improves the delivery of radioactivity to the targeted tissue, which leads to improved therapeutic efficacy. In such drug delivery systems based on nanoparticles, high radiolabeling yield can be achieved due to the high surface area-to-volume ratio of nanoparticles. The large surface area of nanoparticles makes them suitable for modification by adding polymers (e.g., polyethylene glycol) and changing their surface properties to improve the stability and pharmacokinetics in vivo. 

It also offers an opportunity to combine imaging, radiotherapeutic, or chemotherapeutic moieties for multimodal tumor imaging and therapy. The unique chemical and physical properties of some nanoparticles, such as magnetization and photosensitizing, provide additional capabilities for the delivery enhancement of the radioisotopes in the presence of an external magnetic field. The improvement in the therapeutic efficacy can be achieved through the use of near-infrared radiation (Au nanoparticle) or alternating magnetic field-induced hyperthermia [17].

Another advantage of using nanoparticles in Auger electron therapy is their ability to passively accumulate in tumors following systemic intravenous injection via the enhanced permeability and retention (EPR) effect [18]. Because of the rapid vascularization required to serve rapidly growing tumors, most malignancies have leaky vasculature and defective vascular architecture. This fact, in combination with poor lymphatic drainage, allows for increased permeation and retention effects. Because of higher porosity and permeability, the leaky vasculature serves as a natural high-affinity target to various nanoparticles. Unfortunately, local drug deposition is unfeasible for larger tumors with poor vascularization and floating cancer cells such as lymphoma and leukemia. Target specificity is then achieved through the hybrid nanoparticles produced by conjugating them with tumor-specific biomolecules, including mAbs, aptamers, peptides, or various receptor-specific substrates.

Additionally, nanoparticles can avoid uptake by the reticuloendothelial and mononuclear phagocytic systems. The small size of nanoparticles also facilitates their circulation for prolonged retention (especially when polymer-stabilized conjugates are used), compared to compounds with low molecular weight. The ability of some nanoparticles to permanently bind radionuclides, such as gold nanoparticles, which bind heavy halogens such as the ^125^I radionuclide [19,20], is a significant advantage.

In recent years, the concept of delivering radionuclides through nanoparticles for cancer treatment has been highly debated. A number of reviews summarizing studies with the therapeutic application of nanostructures as platforms for β^-^ and α radiation emitters have been published in more than 20 publications [21,22,23,24,25]. To the best of our knowledge, only one review article, a small part of which was devoted to the use of nanomaterials as carriers for Auger electron emitters, has been published so far [26]. 

**Figure 2:**
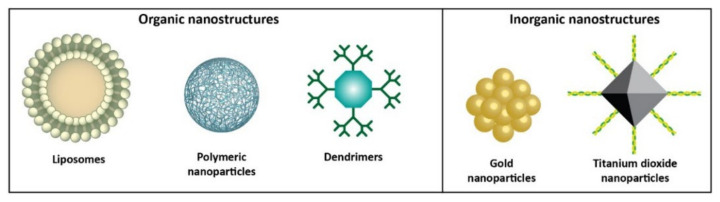
Organic and inorganic nanomaterials discussed in this review.

In this review, the development of nanostructures for Auger electron cancer radiotherapy is analyzed (Figure 2). The current limitations of Auger radiation therapy, such as the requirement to deliver a therapeutic dose directly to the tumor cells, are also discussed. Another purpose of this article is to identify how different types of nanostructures can be used to overcome these limitations. Finally, potential future research opportunities and current questions that must be answered before nanoparticle-based Auger electron radiotherapy can be moved to clinical applications are listed. 

In Auger electron therapy, nanoparticles are primarily used as sensitizers for external beam radiation (the interaction of photons with high-Z nanoparticles, resulting in the emission of low-energy and short-range conversion and Auger electrons, which in turn increase the dose deposited in tissues) or as carriers for the delivery of Auger radiation emitters. Since the use of high-Z element nanostructures as sensitizers has been described in many review articles [27,28,29,30,31], we have limited our review only to radiobioconjugates where nanostructures are used as carriers for Auger electron emitters or as radionuclide carriers that induce the emission of photoelectrons from the nanoparticle surface.

## 2. Organic Nanocarriers

Recently, monoclonal antibodies (such as trastuzumab) targeting the human epidermal growth factor receptor (HER2) have become increasingly important in the treatment of HER2+ breast cancer. Unfortunately, such treatment is ineffective in many cases, including tumors that were sensitive during primary treatment and acquired resistance over time. Trastuzumab–chemotherapeutic conjugates were developed to overcome these limitations. In these conjugates, the chemotherapeutic agent is targeted and internalized by receptor-mediated endocytosis after trastuzumab binding to the cancer cell [32]. One of them is emtansine (DM1) (trastuzumab covalently bonded to the cytotoxic agent DM1), which is commonly used for the treatment of HER2+ metastatic breast cancer (BC) resistant to trastuzumab. Enhancement of therapeutic efficacy is related to the fact that, apart from inhibition of HER2, after prior release of emtansine in the lysosome, the depolymerization of tubulins leads to the prevention of microtubule formation [33]. Another approach for this purpose could be the application of trastuzumab labeled with corpuscular radiation emitters such as α, β, or Auger electron emitters. 

An essential challenge in the production of radiopharmaceuticals based on Auger and conversion electron emitters is to increase their specific activity. Attaching numerous chelators to bigger particles, such as polymers, is a popular approach employed by many researchers. The use of metal-chelating polymers (MCP) with multiple chelating groups to increase the specific activity of the radioimmunoconjugates was first suggested by Torchilin [34], who attached multiple DTPA radionuclide-chelating groups to the amine pendant groups of polylysine. Subsequently, it was found that the maximum specific activity increased linearly with the number of metal chelators attached to the polymer backbone in a series of polyacrylamide-based MCPs with various numbers of attached DTPA and various degrees of polymerization [35].

The application of block copolymer micelles (BCM) for the multiplication of ^111^In transport to cells is described in another publication of this research group [36]. BCMs are nanostructures of amphiphilic copolymers containing a hydrophobic core surrounded by a hydrophilic corona. Therapeutic agents can be encapsulated in the core of the micelles, while the hydrophilic blocks within the corona can be labeled with radionuclides for imaging or radiotherapy [37]. The researchers synthesized a radiobioconjugate containing a 30 nm BCM labeled with Auger electron emitter ^111^In through the DTPA chelator and loaded with (1) radiosensitizer methotrexate, (2) trastuzumab fab vectors for active targeting of HER2+ cells, and (3) nuclear localization signal (NLS; CGYGPKKKRKVGG) peptides for nuclear translocation. The radiosensitizer (methotrexate) was encapsulated within the hydrophobic core of the micelles, while DTPA and NLS-trastuzumab were attached to the fab surface. Cellular accumulation, subcellular localization, and cytotoxicity of the radiobioconjugate were evaluated with MDA-MB-361 and SK-BR-3 human breast cancer cell lines overexpressing HER2 receptors, as well as with MDA-MB-231, which has HER2-negative status. The conjugation of NLS-trastuzumab fab to the surface of BCMs was found to have no effect on the immunoreactivity of the antibody toward HER2+ breast cancer, and it allows uptake of radiobioconjugates into the cell nucleus. The BCM also allows the co-delivery of low concentrations of methotrexate as a radiosensitizing agent. A synergistic cytotoxicity effect through the combining of ^111^In-NLS-trastuzumab fab-BCMs and methotrexate was observed. As a result, it has been demonstrated that the use of BCMs allows for the simultaneous delivery of the Auger electron emitters and the sensitizer molecules to the cell nucleus, significantly enhancing the therapeutic effect.

Next, Reilly et al. [38,39] proposed the use of an MCP containing multiple DTPA molecules in order to increase the amount of delivered Auger emitters. They synthesized three different types of well-defined polyglutamide-based MCPs with hydrazide end groups. DTPA was used as a chelator in each of the pendant groups. In addition to the DTPA ligands, one MCP series contained about four NLS peptides. These polymers were site-specifically conjugated to aldehyde groups generated by NaIO_4_ oxidation of the pendant glycan in the Fc domain of trastuzumab. The obtained immunoconjugates were radiolabeled with ^111^In. Receptor affinity studies demonstrated that neither the MCPs nor the presence of the NLS peptides interfered with specific antigen recognition on SK-BR-3 cells, although nonspecific binding was increased by polymer conjugation. The dissociation constant (K_D_) values were in the low nanomolar range for each polymer. However, this work did not investigate the internalization of these immunoconjugates by SK-BR-3 HER2 receptors or the effect of the attachment of NLS peptides around the cell nucleus.

In previous works of this research group, trastuzumab was modified with DTPA for complexing Auger electron emitter ^111^In and with NLS peptide to direct the delivery of radioimmunoconjugate to the nucleus of HER2-positive BC cells [40]. In in vitro experiments, ^111^In-DTPA-NLS-trastuzumab radiobioconjugate was six times more cytotoxic than non-labeled trastuzumab towards HER2-overexpressing SK-BR-3 human BC cells. Despite these promising results for ^111^In-DTPA-NLS-trastuzumab, a significant limitation was the low specific activity (0.24 MBq/μg) of the radiobioconjugate. As mentioned by the authors, only 1 in 50 molecules of trastuzumab were radiolabeled at such specific activity, resulting in the large numbers of HER2 receptors with unlabeled immunoconjugates, limiting the cytotoxic effect. To significantly increase the specific activity of the radiobioconjugate, the authors decided to label the trastuzumab vector with ^111^In via a metal chelating polymer (MCP) containing multiple molecules of DTPA ligand [39]. The MCP contained a polyglutamide backbone with 29 pendant DTPA (17.4 kDa) or 24 pendant DTPA (23.6 kDa) attached, as well as three NLS peptides (CGYGPKKRKVGG). The MCPs were site-specifically linked to sodium metaperiodate-oxidized glycans on the Fc-domain of trastuzumab to minimize any steric inhibition of the MCP on binding to HER2 receptors. The obtained specific activity of ^111^In labeled MCP-trastuzumab (8.9 MBq/μg) was 90 times greater than that of ^111^In labeled trastuzumab modified with only two DTPA groups. Indium-111 labeled MCP-trastuzumab was bound, internalized, and transported into the nucleus of SK-BR-3 cells, but the process was slower than in the case of ^111^In labeled trastuzumab. The nuclear uptake was more significant for the polymer radioimmunoconjugates, but NLS peptide modification did not improve the transport to the cell nucleus. As HER2 receptor internalization is lower than other members of the EGFR family, only 10–20% of ^111^In labeled MCP-trastuzumab incubated with breast cancer cells was internalized (which was 30–50% of the radiobioconjugate bound to the cells), and most of the radiobioconjugates remained on the cell membrane. However, membrane-located Auger electron emitters have been shown to cause cytotoxicity in cancer cells [41]. The authors concluded that increasing the specific activity by conjugation of MCPs to trastuzumab greatly amplified the cytotoxic potency against HER2-overexpressed breast cancer cells and extended its cytotoxicity to cells with intermediate HER2 expression but without gene amplification, as well as to cells that are HER2 overexpressed but trastuzumab-resistant.

The influence of the type and construction of the polymer on biodistribution and cellular uptake of MCP-mAb conjugates is an essential issue in the design of MCP-mAb conjugates for clinical applications. The pendant groups can also play an important role. Boyle et al. [42] synthesized a series of polymers having a biotin end group to simplify the construction of MCP conjugates. In parallel, they derivatized a fragment of trastuzumab with streptavidin using a poly(ethylene glycol) linker. Streptavidin is a 66 kDa protein with a strong biotin affinity. Taking into account the dissociation constant (10^−14^ mol/L), the non-covalent bond between streptavidin and biotin is considered one of the strongest found in nature. This one-of-a-kind characteristic is the main reason why it is an ideal candidate for several medical applications, including drug delivery systems. Moreover, this allows for the simple functionalization of streptavidin with biologically active biotinylated molecules. It was shown that the attachment of the MCP to the trastuzumab-streptavidin conjugates had no significant effect on the rate of binding to the extracellular domain of HER2 or the dissociation rate (the interaction was characterized by a low nanomolar binding constant). The biodistribution and micro SPECT/CT (single-photon emission computed tomography/computed tomography) imaging studies on Balb/C mice showed that the tissue distribution of the trastuzumab Fab-streptavidin-MCP conjugates was dependent on both the chemical structure of the polymer backbone and the nature of the pendant group to which the DTPA units were attached [43]. Rapid elimination from the blood and high liver uptake have been found to be related to the polyanionic character of the MCP. In the absence of In^3+^ saturation, there were several negative charges on each pendant group. When the polymer was saturated with In^3+^, the DTPA^4-^-In^3+^ complex had a charge of -1. As a result, the indium-saturated trastuzumab Fab-streptavidin-biotin-MCP conjugate was an anionic polyelectrolyte, as well. However, the ethylenediamine diamide was used as a linker between MCP and DTPA instead of the diethylenetriamine diamide, which contains a secondary amine protonated at neutral pH. Polymers with diethylenetriamine diamide-DTPA pendant groups are zwitterionic when saturated with trivalent metal ions. In this space at neutral pH, the negative charge of the DTPA^4-^-In^3+^ complex is balanced by the positive charge associated with the protonated secondary amine of the diethylenetriamine diamide. It is known that the incorporation of zwitterionic polymers into micelles and other colloidal particles can reduce protein adsorption to surfaces and enhance blood circulation time. The authors concluded that the MCPs with a neutral net charge are the most suitable for the construction of radioimmunoconjugates. Unfortunately, due to the immunoreactivity of streptavidin and enhancement of streptavidin uptake by the kidneys, these conjugates are not acceptable for clinical use. Nevertheless, they are effective preclinically as a rapid method of examining site-directed MCPs.

Despite this, studies on streptavidin-modified nanoparticles were continued by the Hnatowich group [44]. They synthesized streptavidin-based nanoparticles, which were functionalized with two or three biotinylated components. In this concept, they functionalized streptavidin-based nanoparticles with three components: trastuzumab for effective HER2 receptor targeting, tat-peptide (containing NLS sequence) for the enhancement of cell membrane and nuclear envelope penetration, and antisense morpholino oligomer (MORF) for further targeting of RNA or DNA in the nucleus. Morpholinos are oligomers containing a methylenemorpholine ring as a framework, bound to each other with phosphorodiamidate groups. In general, MORF consists of about 25 of these sequences, each of them containing one of the DNA bases, enabling them to interact with ssDNA and mRNA. MORF and other oligomers can be designed as sense and antisense structures. The sense transcript is arranged from non-coding (antisense) DNA, while the antisense transcript is complimentary to coding (sense) DNA. Obviously, during transcription, when RNA is formed from a DNA matrix, thymine is replaced by uracil.

The biological function of MORF is to regulate gene expression, for example, by sterically blocking RNA access to molecules that could interact with RNA. According to this, MORF was considered a promising targeting agent. For this purpose, Liu et al. [45] investigated the possibility of MORF radiolabeling with ^90^Y and ^188^Re (β^-^), ^111^In (Auger), and ^99m^Tc (γ) using DOTA (Macrocyclics, Dallas, TX, USA) or NHS-MAG_3_ chelators and then performed stability and biodistribution studies in normal mice. Radiolabeling of MORF-DOTA with ^111^In was performed with a 60% yield. After P4 purification, specific activity ranged from 60 to 90 µCi/µg, with a final purity of >93%. Stability studies in saline and human serum (HS) confirmed 48 h stability of ^111^In-MORF in contrast to ^90^Y-MORF, which showed a decrease in stability due to radiolysis and ^90^Y-DOTA release. During biodistribution studies in normal mice, any unexpected accumulation in organs was noticed. Only the renal clearance mechanism revealed higher percentages of 9.31% and 8.38% ID/g in the kidneys after 1 and 3 h, respectively.

The higher accumulation of MORF in healthy tissues compared to cancerous tissues is a significant disadvantage of MORF systemic application in cancer treatment. When added to this also limited internalization, using them with radioactive isotopes becomes a challenge. To overcome these limitations and improve the pharmacokinetics, the same group [46] proposed MORF conjugates with protein nanoparticles. The general concept was to use streptavidin as a carrier molecule, conjugated with biotinylated (1) MORF, (2) monoclonal antibody, and (3) tat-peptide. They initially demonstrated that biotinylation had no effect on the biological functions of the used components. During primary studies, ^111^In was used due to its accessibility and prior successful ^111^In-MORF radiolabeling. Subsequently, evaluation of the cell and nucleus penetration ability against mRNA and verification of the cytotoxic potential of Auger emitter ^111^In were evaluated. In some of the studies, ^99m^Tc-antisense/tat/trastuzumab and ^99m^Tc-sense/tat/trastuzumab radiobioconjugates were used for subcellular and in vivo biodistribution calculations. The percentage uptakes of ^99m^Tc-antisense/tat/trastuzumab in the cells and nucleus were 8.20 ± 0.38% and 7.62 ± 0.42%, respectively. In comparison, the cell and nucleus fractions of ^99m^Tc-sense/tat/trastuzumab were 7.20 ± 0.30% and 6.54 ± 0.34%, respectively. Cytotoxicity studies confirmed ^111^In-related cell survival decrease for both antisense/sense fragments at a dose of 2.04 MBq/well. The membrane integrity assay yielded some interesting results, with the authors discovering that unlabeled nanoparticles combined with ^111^In-DTPA were more efficient than radiolabeled nanoparticles bioconjugates. This was explained as a consequence of the synergistic interaction of the antibody and extracellularly located Auger emitter with the cell membrane. Dose escalation studies showed that as radioactivity increased, the surviving fraction decreased, with negligible non-specific toxicity. The cell survival fraction assay differentiated antisense and sense conjugates, with antisense nanoparticles showing a greater reduction with increasing radioactivity than sense nanoparticles. This was probably related to the enhanced nuclear uptake of antisense oligomers. Animal studies using ^99m^Tc-antisense/tat/trastuzumab revealed the typical and extensively reported problems associated with nanoparticles. In addition to the tumor site (10.45% ID/g), high uptakes were also noticed in the liver (10.4% ID/g), kidneys (19.7% ID/g), and spleen (5.09% ID/g). Immunohistochemistry observations showed a lack of accumulation of tested bioconjugates in the nucleus of normal tissue after the intravenous application. In general, in vitro studies proved the high potency of streptavidin-based antisense/tat/trastuzumab nanoparticles, including cytotoxicity, and confirmed specific and rapid intranuclear location in cancerous cells, especially for antisense conjugates. The in vitro results proved that the antibody, despite its size, can also penetrate the nuclear envelope when the NLS peptide is used. The authors also concluded that both ^111^In and MORF were responsible for the cytotoxic effect. However, in vivo biodistribution remains a crucial limiting parameter.

The same radiobioconjugates were evaluated using ^125^I instead of ^111^In in subsequent experiments, as described in another publication by Hnatowich [47]. In comparison to ^111^In (t_1/2_ = 2.8 d), which emits eight Auger electrons per decay, ^125^I emits 21 Auger electrons per decay and has a much longer half-life (t_1/2_ = 60 d). The lack of gamma emission and potential instability due to dehalogenation, common in halogenated chemicals in biological systems, are both disadvantages of ^125^I, as reported by the authors. Tyrosine residues were used for ^125^I incorporation into the MORF. A direct comparison of the results presented in both publications [46,47] is challenging due to the fact that the SK-BR-3 cell line was utilized for the ^111^In evaluation, and the BT474 cell line was used for the ^125^I evaluation. This is doubtful, especially since BT474 cells are thought to be more radiosensitive. Additionally, as previously stated, the half-lives of both radionuclides are significantly different. However, the minimum dose inducing cytotoxicity for ^125^I was three times lower than for ^111^In (10 µCi/well vs. 30 µCi/well). The authors hypothesized that a significant nucleus-to-cytoplasm ratio might be the result of non-covalent MORF binding to streptavidin and related to this improved release of, e.g., MORF. Even when the K_D_ parameter for streptavidin-biotin bound is set to a high value, it is still weaker than the covalent bound. 

To verify this concept, they performed analogous studies as reported in the publication [48]. In these studies, the effects of two compounds were compared. Streptavidin-based “non-covalent” compounds consisted of Cy3-MORF/tat/trastuzumab. “Covalent” compounds included Cy5.5-DNA-trastuzumab (for fluorescent imaging) and ^111^In-DTPA-trastuzumab-antisense DNA (for radiometric assays). Flow cytometry analysis confirmed high accumulation of the tested bioconjugates only in the case of HER2+ SK-BR-3 cells, at a similar level as native trastuzumab. Fluorescent imaging proved that both non-covalent and covalent conjugates were localized in the cytosol. However, non-covalent conjugates were also found in the cell nucleus after 3 h of incubation. Radiometric subcellular distribution assays once again confirmed specific binding to HER2 receptors and the lack of internalization in HER2- MDA-MB-231 cells. The authors concluded that the presence of a covalent bond was responsible for the reduced release. 

In a recently published work, Qin et al. [49] developed a curcumin-loaded nanomicelle composed of a photosensitizer chlorin e6 (Ce6) and amphiphilic poly(ethylene glycol) (poly(maleic anhydride-alt-1-octadecene)-poly(ethylene glycol) (C18-PMH-PEG)) to deliver ^125^I to the nucleus, followed by 660 nm laser irradiation. These nanomicelles were designed for image-guided internal Auger and conversion electron therapy of cancer. The nuclear uptake of ^125^I-labeled curcumin containing a phenolic hydroxyl group was observed under confocal microscopy and was consistent with previously published work [50]. The tumor was detected by fluorescence and SPECT imaging two hours after intravenous injection of ^125^I-labeled Ce6-C18-PEG/Cur nanomicelles. The tumor was then irradiated with a 660 nm laser under bimodal imaging. The photodynamic reaction to the chlorin e6 photosensitizer enhanced the effect of EPR by destroying tumor blood vessels, increasing the cellular uptake of ^125^I-labeled Ce6-C18-PEG/Cur and promoting lysosome escape of the nanoparticle. The released ^125^I-labeled Cur was subsequently transported into the nucleus, where it caused DNA double-strand break damage in both in vitro and in vivo conditions. The presented studies demonstrated a novel strategy for the effective treatment of cancer that involves the delivery of Auger electron emitters into the cell nucleus under the local laser irradiation. 

In another study, nanoparticles prepared from the widely used chitosan biopolymer were applied for nanoparticle-mediated radionuclide-gene therapy of liver cancer [51]. The alpha-fetoprotein (AFP) with the antisense oligonucleotide was labeled with Auger emitter ^125^I and encapsulated with chitosan nanoparticles. The nanoparticles were then transfected into liver cancer cells (HepG2 cells) to disrupt the expression of their AFP gene. The damages of DNA and proteins in cells were determined by using the nanoscale confocal Raman scattering microscope (LabRam INV, Kyoto, Japan). The expression of the AFP gene and protein were also observed. All the biological effects were measured as the functions of the radiation intensity, the time of the transfection, and the size of the nanoparticle. The Auger electron generated by ^125^I was discovered to damage the helical conformation and structure of DNA and depress the AFP gene expression. The DNA damage increased with the radiation intensity. The authors found that antisense oligonucleotide is an effective specific carrier of the Auger emitter ^125^I into the target DNA, with chitosan nanoparticles inducing two times more damage than the pure ^125^I-AFP-antisense oligonucleotide.

Other essential radionuclide nanocarriers, particularly Auger electron emitters, are dendrimers [52]. They have been widely studied as carriers of β^-^ and α particle emitters, and two papers on their application as Auger electron emitters transporters have been published [14,53]. Dendrimers are unique among nanomaterials because of their stepwise synthesis, which enables the formation of well-defined and monodisperse structures with tunable size and number of terminal units. They consist of three main structural elements: the inner core from which dendritic branches grow, branch layers that define the generation of dendrimers, and a multivalent outer shell. The number of peripheral groups in dendrimers increases exponentially with the generation number [46]. This allows for the attachment of a large number of chelating molecules that can be labeled by cations for MRI, fluorescence, CT, and radionuclide-based imaging and therapy. The regulated structure of dendrimers also enables the simultaneous attachment of chemotherapeutic molecules and targeting moieties. The most popular is the PAMAM (poly(amidoamine)) dendrimer, which is constructed of repetitively branched amide and amine functional subunits. PAMAM dendrimers have been used to attach several chelators and targeting vectors because of their vast surface amine groups. Due to the polycationic surface, the electrostatic interaction with negatively charged residues on the cell surface is believed to trigger the endocytosis process. Mamede et al. [54] synthesized PAMAM dendrimer with avidin radiolabeled through DTPA chelator with a very high specific activity of Auger and conversion electron emitter ^111^In. They evaluated its internalization, biodistribution, and therapeutic effect in mice with intraperitoneal disseminated tumors. Avidin is a basic glycoprotein with a molecular mass of 67 kDa, which possesses a high-affinity biotin-binding site. Since the degree of complexation of chelating sites can affect the behavior of the radiolabeled compound, the DTPA chelating sites on bioconjugate were saturated with either radioactive or non-radioactive In^3+^. The final product contained 52 DTPA chelator molecules linked to a single G4 molecule. It should be possible to achieve extremely high specific activity (theoretically, 37 GBq/µg). The radiolabeled dendrimer was internalized to SHIN-3 cells at 37 °C, and 77.6% of the radioconjugate was internalized after 24 h of incubation. The results clearly show the ability of this compound to internalize into cancer cells specifically, although its intracellular localization has yet to be discovered. The biodistribution in mice bearing an intraperitoneal disseminated tumor demonstrated a high dendrimer concentration in disseminated tumors with high tumor to background ratios.

In a more recent study, generation 1 and 4 PAMAM dendrimers were conjugated with a bifunctional pyridine-N-oxide DOTA analog and radiolabeled with Auger electron emitter ^111^In [55]. The conjugate displayed satisfactory kinetic stability for at least 48 h after preparation. In the presence of EDTA/DTPA ligand competitors, the stability of the conjugates slightly decreased over the same time period. Biodistribution and elimination in rats were more favorable for the generation 1 ^111^In conjugate than for generation 4. While the generation 1 ^111^In conjugate was rapidly eliminated from the body, mainly through the urine, generation 4 ^111^In showed high and long-term radioactivity accumulation in the liver and kidney, and this limits the use of this dendrimer in nuclear medicine.

**Figure 3:**
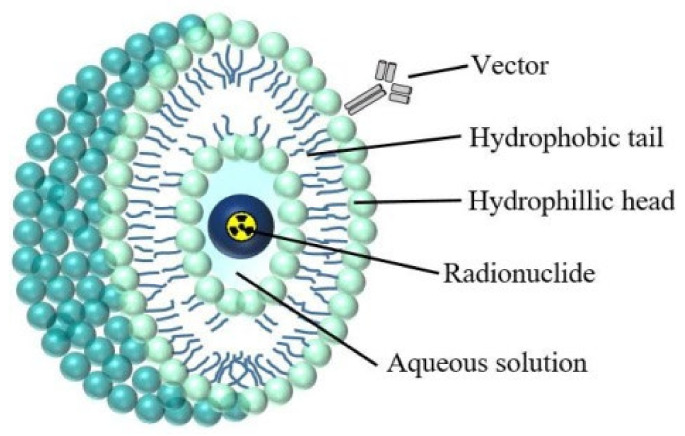
Structure of a liposome containing a radionuclide [25].

Liposomes (Figure 3) are a class of nanoparticles that are well suited to cancer therapy due to their ease of production and ability to encapsulate a large payload of active therapeutics within the aqueous core of their spherical lipid bilayer. Hydrophilic substances dissolved in the core are unable to pass through the bilayer membrane easily [56]. Additionally, liposomes can be surface-modified with polyethylene glycol to protect them from the reticuloendothelial system (RES) and attach a targeting vector. 

Liposome drug delivery systems have shown significant success in cancer therapy, with various products approved for use. Doxil^®^, a pegylated liposome containing doxorubicin, was the first FDA-approved nanodrug in 1995. Doxil^®^ liposomes have been found to accumulate preferentially in mouse model tumors [57] as well as in patients with primary and metastatic disease [58].

Fondell et al. developed a two-step targeting strategy to meet the above criteria for effective targeted radiotherapy using Auger electron emitting radionuclides [59]. This concept was based on the use of liposomes loaded with significant amounts of ^125^I-labeled amino-benzyl derivative of daunorubicin, which facilitates the localization of the internalized radionuclides in close vicinity to DNA in the cell nucleus [60]. The liposomes were also functionalized with anti-HER2 single-chain fragment F5 as a targeting agent. HER2+ tumor cells from both ovarian and breast cancer cell lines demonstrated rapid uptake of ^125^I-labeled daunorubicin when delivered by F5-targeting liposomes. Biodistribution studies suggested that the developed bioconjugate (^125^I labeled daunorubicin inside the functionalized liposome) accumulated highly in cancer cells compared to non-targeting liposomes. Hence, HER2-targeting liposomes loaded with the ^125^I- amino benzyl by-product of daunorubicin are cell-toxic and also possess suitable therapeutic potential. Unfortunately, there have been no in vivo toxicity investigations.

In another research paper, tumor targeting and diffusion efficiency of the most clinically used liposomes, multilamellar vesicles (MLVs), small unilamellar vesicles (SUVs), and anti-PSMA monoclonal antibody (mAb) J591 studied on prostate cancer cell spheroids have been described [61].The most heterogeneous distribution of the absorbed dose was achieved with Auger or conversion electron emitters (^123^I, ^125^I); the absorbed dose ratios at the center of the spheroid (D_core_) to the maximum absorbed dose (D_max_) were 0.40 and 0.38 at 28 and 26 µm depth for the SUV-DMPC-chol liposome, respectively, and 0.42–0.52 for the antibody. At the center of the spheroid, the absorbed dose was increased by 2 to 10 times on average (Dcore). However, Auger emitters delivered more than 40% D_max_ to the (3β-[N-(N′,N′]-dimethylaminoethane)carbamoyl]cholesterol [DC-chol])—liposomes with high binding capacity, compared to β^-^ emitters. According to this study, it is possible to design radiolabeled liposomes capable of delivering a homogenous absorbed dose to the core of micrometastatic tumors.

Recently, Owen et al. [62] proposed the application of ultrasound-induced cavitation to release ^111^In-labeled bioconjugates from liposomes. The liposome was loaded with a peptide, human epidermal growth factor (hEGF), coupled to a chelator for subsequent radiolabeling with indium-111. The obtained liposomes were efficiently radiolabeled with a 57% yield within 1 h. In the in vitro studies, the exposure of liposomes to ultrasound-activated cavitation for 20 s resulted in the release of ~12% of the ^111^In labeled peptide. In biodistribution studies, it has also been demonstrated that the majority of ^111^In-hEGF was accumulated in the kidneys and liver, whereas for liposomes loaded with ^111^In-HEGF, renal clearance was reduced by 50%. Surprisingly, there was no significant accumulation of the loaded liposome in the liver. This study presented a novel approach to the targeted delivery of radiopharmaceuticals by combining acoustic cavitation-sensitive liposomes with radionuclides. In the conducted studies, the emission of γ quanta by ^111^In was used to test biodistribution; however, the therapeutic properties of the radiopharmaceutical related to the emission of Auger electrons were not investigated.

## 3. Inorganic Nanocarriers

Inorganic nanoparticles, due to their size, chemical, and physical properties, are particularly attractive as therapeutic probes in cancer treatment. Radionuclides with specific emission properties can be incorporated into nanoparticles or attached to their surface and used for radionuclide therapy and radio imaging. Among the large number of synthesized inorganic nanoparticles, only gold nanoparticles (AuNPs) have been widely used as radionuclide carriers in Auger electron therapy, and in single studies, platinum and titanium oxide nanoparticles were also evaluated.

**Figure 4:**
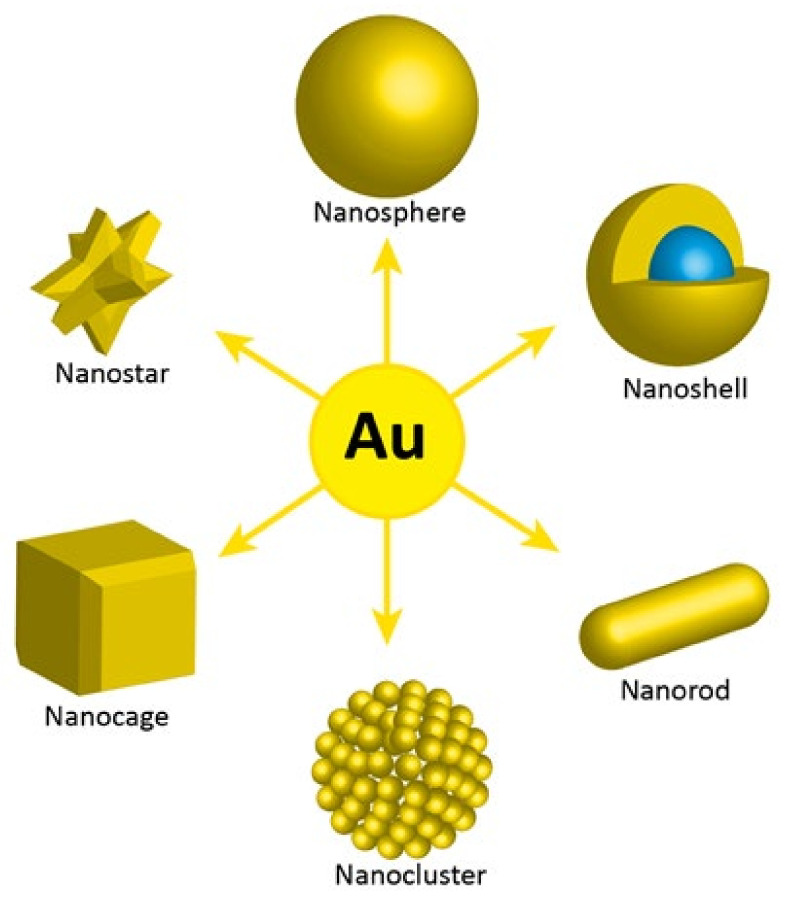
Different types of AuNPs, according to their shape and morphology.

The unique properties of AuNPs, including surface properties, allow their use in a number of therapeutic methods where direct or indirect attachment of radionuclides is required. Additionally, AuNPs offer the possibility of surface functionalization with targeting biomolecules for specific accumulation and interaction with receptors overexpressed in tumor cells. Moreover, various synthesis methods are now available to manipulate the size and shape of gold nanoparticles. The following gold nanostructures have been synthesized and studied for different medical applications: nanospheres, nanorods, nanocages, nanostars, clusters, and core shell nanoparticles (Figure 4). 

Several studies have shown [63,64,65] that gold nanoparticles can be very effective in promoting the cytotoxic effect towards cancerous cells such as melanoma or head and neck tumors very effectively during irradiation with 150–250 kV X-rays. The results of these studies are noteworthy, as the administration of AuNPs followed by X-ray treatment reduced the size of the tumors and, in some cases, destroyed tumors significantly. Because AuNPs can be visualized through CT and planar X-rays, these nanoparticles, if systematically targeted, could serve as dual imaging (diagnostic) and therapeutic probes in the detection and therapy of cancer (theranostics) [66]. The potential of AuNPs in theranostics was also described by Chanda et al. [67]. The radioactive decay properties of ^198^Au (t_1/2_ = 2.7 d, β_max_ = 0.96 MeV) and ^199^Au (t_1/2_ = 3.14 d, β_max_ = 0.46 MeV) make them promising candidates for therapeutic applications. In addition, they both emit gamma quanta for dosimetry and pharmacokinetic studies. In studies related to Auger electron therapy, AuNPs are used as carriers for Auger electron emitters such as ^125^I, which can be attached directly to the gold surface, or ^111^In, which is complexed via DOTA or DTPA chelators. The nanoparticles were also surface-modified with monoclonal antibodies or peptide ligands to bind to tumor-associated antigens or receptors for active tumor targeting. 

Radiobioconjugate biodistribution studies were initially conducted for future use in X-ray-induced radiotherapy. Reilly et al. synthesized radiobioconjugates by the modification of gold nanoparticles (30 nm) with polyethylene glycol chains linked to trastuzumab to target HER2-positive breast cancer (BC) cells and DTPA chelator to complex ^111^In [68,69]. These trastuzumab-AuNP-^111^In conjugates were further surface-coated with PEG to stabilize the particles against aggregation and minimize liver and spleen uptake. Despite long-chain PEG modification, tumor uptake in athymic mice with HER2-overexpressing MDA-MB-361 human BC xenografts was limited (1.2% ID/g) at 48 h post-intravenous injection. The uptake of trastuzumab-AuNP-^111^In in the liver was low in contrast to uptake in the spleen (3% ID/g and 20% ID/g, respectively). Sequestration of AuNP by the liver and spleen, resulting in rapid clearance from the circulation and limited tumor uptake, is thus a major restriction of the intravenous administration of AuNP. The authors investigated local intratumoral injection of the bioconjugate to avoid liver and spleen uptake and maximize tumor localization of trastuzumab-AuNP. This route of administration yielded a 25-fold increase in trastuzumab-AuNP-^111^In concentration in the tumor after 48 h p.i. (30% ID/g) and a 10-fold decrease in the spleen uptake (2% ID/g). Therefore, intratumoral injection was used for further studies on the therapeutic effects of the trastuzumab-AuNP bioconjugates. This method should have clinical application as a form of neoadjuvant brachytherapy in which nanometer-sized seeds are bound and internalized into tumor cells, increasing the effectiveness of the therapy. Although trastuzumab-AuNP enhanced the effectiveness of X-radiation treatment of MDA-MB-361 tumors, the radiosensitizing properties were moderate [70].

As highlighted in Table 1, ^111^In (t_1/2_ = 2.8 d) emits an average of 6.9 low-energy Auger electrons as well as γ quanta (Eγ = 171 keV (90%) and 245 keV (94%)). Therefore, it is one of the most promising Auger electron emitters. Previously, Reilly’s team [40] reported on ^111^In-labeled trastuzumab containing a nuclear translocation sequence (NLS) peptide. Effective HER2-targeting SKBR-3 cells and subsequent—due to NLS presence—intranuclear location caused multiple DNA double-strand breaks, resulting in a 90% reduction in the survival of SKBR-3 breast cancer cells. As mentioned before, the obtained specific activity of labeled trastuzumab (0.24 MBq/μg) is insufficient for successful Auger therapy [14]. Therefore, as in the case of the previously described application of the chelating block copolymer to multiply ^111^In transport to cells [36], the authors decided to use AuNPs to multiply ^111^In transport to cells. The in vitro cytotoxicity of trastuzumab-AuNP-^111^In on HER2+ breast cancer cells and their in vivo studies on tumor growth inhibitory properties and tissue toxicity after intratumoral injection in athymic mice with s.c. (injected subcutaneously) HER2-overexpressing MDA-MB-361 human BC xenografts have been described by Cai et al. [71]. The researchers showed that trastuzumab-AuNP-^111^In was bound to and internalized by HER2+ human breast cancer cells (SK-BR-3 and MDA-MB-361). Unfortunately, the determined K_D_ for binding trastuzumab-AuNP-^111^In to the HER2 receptor on SK-BR-3 cells was 46 times lower than previously reported for ^111^In-labeled trastuzumab. The reduced binding affinity of trastuzumab-AuNP-^111^In can be caused by a steric hindrance of AuNPs to HER2 receptors and a large number of trastuzumab and PEG molecules attached to each AuNP, which may further contribute to steric inhibition of receptor binding. 

However, a greater number of trastuzumab molecules on AuNP provides stronger multivalent binding of trastuzumab-AuNP-^111^In bioconjugate to breast cancer cells in comparison to ^111^In-trastuzumab alone. The authors stated that a lower K_D_ value for trastuzumab-AuNP-^111^In can most likely be achieved by reducing the number of trastuzumab and PEG molecules attached to AuNP, reducing steric hindrance in HER2. 

Cellular studies have also shown that internalization was more efficient for trastuzumab-AuNP-^111^In than with AuNP-^111^In. As it is well known [72], trastuzumab binding promotes HER2 internalization. Widefield microscopy in fluorescence and dark-field modes revealed that trastuzumab-AuNP-^111^In was internalized and deposited near the nucleus in the perinuclear area of SK-BR-3 and MDA-MB-361 cells. In contrast, the internalization of ^111^In-labeled AuNPs was not visible. As trastuzumab locates in breast cancer cells in liposomes close to the nucleus, this affects the ability of the emitted Auger electrons to cause lethal DNA double-strand breaks. Since lysosome uptake of trastuzumab degrades HER2 receptors, resulting in cell death, the authors hypothesized that trastuzumab-AuNP-^111^In might be toxic to breast cancer cells in two ways, by reducing HER2 and by causing DNA damage in breast cancer cells, owing to Auger electron emission.

In vivo studies were performed by local intratumoral injection of trastuzumab-AuNP-^111^In in CD1 (cluster of differentiation 1) athymic mice with s.c. MDA-MB-361 xenografts that stopped tumor growth over 70 days. It was found that the injection of trastuzumab-AuNP-^111^In had no effects on body weight, liver damage, renal toxicity, or hematopoietic toxicity compared to normal saline-treated control mice at 70 days. Irradiation of hematopoietic bone marrow was minimal due to intratumoral injection and the short range of Auger electrons. 

In another study, Song et al. [73] described the cytotoxicity of ^111^In-labeled AuNP on BC cells. In this study, AuNPs (14 nm) were conjugated to ^111^In-DTPA-EGF to bind epidermal growth factor receptors (EGFR) on breast cancer cells. EGF-AuNPs were prepared via direct interaction between gold and the disulfide bonds of EGF and then radiolabeled with ^111^In, wherein it was shown that direct attachment of EGF to AuNPs does not perturb EGF-EGFR binding. The obtained results suggested that the disulfide groups of EGF can be exploited for binding to AuNPs. The synthesized ^111^In-DTPA-EGF-AuNP conjugate was bound and internalized by EGFR-overexpressing MDA-MB-468 human breast cancer cells, reducing their surviving fraction to 4.4% after 4 h exposure. Assessment of double-stranded DNA breaks caused by Auger electrons has not been performed in this work.

Another study was conducted to evaluate the possibility of using AuNPs as a carrier for the Auger electron emitter ^125^I [20]. The authors used the high affinity of gold atoms to heavy halogens for the immobilization of ^125^I [74]. This is due to their nearly identical electronegativities, which are 2.4 for gold and 2.5 for iodine. The aim of this study was to use a citrate reduction technique (Turkevich method) for one-step synthesis of AuNPs labeled by ^125^I. Appropriate synthesis conditions were selected to avoid the aggregation of nanoparticles. Synthesized nanoparticles were found to be extremely stable without any observable leakage of radioactivity into solution. 

Apart from gold, TiO_2_ nanoparticles were also applied as carriers for ^125^I [75]. Because of their high surface activity and lack of toxicity, TiO_2_ nanoparticles are widely used in medicine, including cancer therapy, antibacterial treatment, and biomolecule detection. Excess electrons (e.g., photoinduced electrons) are recognized to play an important role in the surface chemical activity of TiO_2_ nanocatalysts. Due to the restricted penetration of light in tissue, the in vivo generation of excess electrons by light is difficult or impossible. Hence, this approach is only suitable for superficial tumors. In original studies, Su et al. [75] used ^125^I to inject Auger electrons into TiO_2_ nanoparticles to create in vivo active sites and investigated its use in cancer treatment. In the synthesized ^125^I-TiO_2_ NPs, Ti^3+^ ions were formed by the reaction between Ti^4+^ and Auger electrons emitted from ^125^I. Consequently, Ti^3+^ stretched the O-H bond of the absorbed H_2_O to decrease its bond energy, facilitating H_2_O radiolysis. Finally, upon irradiation of γ-rays emitted by ^125^I, the radiolysis of activated H_2_O will occur more efficiently, leading to an enhanced generation of •OH radicals. As a result, the therapeutic effect of Auger electrons emitted by ^125^I is combined with the toxic effect of •OH radicals generated on TiO_2_ NPs’ surface in the proposed system. The in vitro and in vivo studies showed high effectiveness of the proposed therapy. The therapeutic effect of ^125^I-TiO_2_ in vivo was examined in tumor xenograft mice. One group of mice received intratumorally free ^125^I and the second group received intratumorally ^125^I-TiO_2_. In the group that received ^125^I-TiO_2_, inhibition of tumor growth was observed compared to the free ^125^I group. Correspondingly, the survival rate in the ^125^I-TiO_2_ group was significantly improved over a 60-day follow-up period, during which all mice in the ^125^I group died. However, this effect could be related to poor ^125^I retention in the tumor and its diffusion to other organs, while the tumor retention of ^125^I-TiO_2_ was high.

## 4. Auger Electron Generation on High Atomic (Z) Number Nanocarriers

Recently, more and more attention has been paid to the use of nanoparticles with a high-Z number to increase the radiation-absorbed dose deposited in tumors from external X-ray radiation. The radiosensitization effect is based on the strong interaction of photons with high-Z atoms, resulting in an increase in the local dose deposition and emission of conversion and Auger electrons. This phenomenon is of great interest to radiation oncologists as it facilitates the generation of secondary electrons when nanoparticles are placed in cancer cells and exposed to X-rays. Several groups have worked on the development of new radiosensitization agents using high-Z elements such as Au (Z = 79), Hf (Z = 72), Gd (Z = 64), I (Z = 53), Pt (Z = 78), and Bi (Z = 83) [30]. As mentioned before, high-Z element nanostructures as sensitizers of external X-radiation have been described in many review articles, and therefore, we limited our review to the systems in which high-Z nanoparticles are used as radionuclide carriers that emit photoelectrons from their surface.

In a pioneering publication, another type of nanoparticle-enhanced radiation therapy was suggested by Pronschinske et al. [76]. They synthesized one-atom-thick layers of the radionuclide ^125^I on gold and reported large amplification of low-energy electron emission. By scanning microscopy, supported by electronic structure simulations, they observed the nuclear transmutation of individual ^125^I atoms into ^125^Te and explained the surprising stability of the 2D film as it underwent radioactive decay. The metal interface geometry induced a 600% amplification of low-energy electron emission compared with atomic ^125^I. This enhancement of biologically active low-energy electrons could indicate a new direction for highly targeted nanoparticle therapies.

Another radionuclide, ^169^Yb, has been proposed to generate photo and Auger electrons on AuNPs. The ^169^Yb (t_1/2_ = 32.0 d) is an ideal radioisotope for this purpose because it emits photons with an average energy of 93 keV (just above the K edge of gold). Due to the absence of a commercially available ^169^Yb source providing a sufficiently high dose rate necessary for in vitro and in vivo studies, the authors developed a surrogate external beam ^169^Yb using erbium (Er) as a filter material and a standard copper (Cu)-filtered 250 kVp beam. They investigated the role of the energy range of photons present in the γ-ray spectrum of ^169^Yb for gold-mediated radiosensitization through in vitro and in vivo studies. Human prostate cancer cells were pre-treated with gold nanoparticles using goserelin-conjugated gold nanorods and non-targeted gold nanoparticles.

Then, in vivo experiments were performed by irradiating tumor-bearing mice (human prostate cancer xenograft) pre-treated with actively targeted and non-targeted nanoparticles. The results revealed that the treatment with goserelin-conjugated gold nanorods in combination with X-ray irradiation (mimic ^169^Yb radiation) is considerably more effective than radiation treatment alone.

Chao et al. [77] applied the ^99m^Tc, a low-energy γ emitter, to generate conversion and Auger electrons by reaction with Hf atoms. Technetium-99m is one of the most widely used radioisotopes in diagnostic imaging, mainly for single-photon emission computed tomography (SPECT). Although ^99m^Tc is the most commonly used radionuclide for imaging, it is only occasionally used for therapy. In a one-pot reaction, the authors prepared a PEG-modified coordination polymer, consisting of Hf^4+^ cations and tetrakis (4-carboxyphenyl) porphyrin, used as a chelating agent. Such a modified coordination polymer was labeled with ^99m^Tc in high yield. The inherent biodegradability of coordination polymer nanoparticles due to weak coordination interactions between metal ions and organic linkers is an advantage compared to the typical inorganic nanoparticles. The in vivo therapeutic efficacy of ^99m^Tc-labeled coordination polymer was evaluated following intravenous systemic administration and intratumoral local injection in mice bearing 4T1 tumors. In vivo SPECT imaging studies indicate that ^99m^Tc-labeled coordination polymer shows prolonged tumor retention after local intratumoral injection as well as tumor accumulation by the EPR effect after intravenous injection. As a result, a significant therapeutic effect has been achieved, substantially delaying tumor growth by a single injection or eliminating tumors by multiple repeated treatments.

## 5. Conclusions

Radionuclide delivery systems using nanoparticles have notable potential in the field of nuclear medicine. In the case of Auger electron therapy, nanoparticles of high-Z elements are commonly studied as sensitizers of external X-ray radiation and have been described in many articles. These studies have now moved to the stage of clinical trials. However, the use of nanostructures as carriers for Auger electron emitters is still limited. This is undoubtedly related to the difficulty of systemic administration, which causes the sequestration of nanostructure conjugates by the liver and spleen, resulting in rapid blood clearance and minimal tumor absorption. Nevertheless, recently proposed novel targeted nanobrachytherapy approaches for the treatment of locally advanced breast and prostate cancers should broaden the application of nanoparticles in Auger electron therapy. The organ distribution studies reveal that intratumorally delivered radiolabeled nanoparticle bioconjugates are almost completely retained in the tumor, having minimal uptake in the liver and spleen. In comparison with classical labeled biomolecules, nanostructures with attached Auger electron emitters have a significant advantage of delivering significantly higher radioactivity to the cancer tissue. This is particularly important in Auger electron therapy, where substantially higher radionuclide activity is required to achieve a therapeutic effect compared to α and β- emitters.

## Figures and Tables

**Table 1:** Characteristics of radionuclides with possible applications in Auger electron targeted therapy. The most promising radionuclides are marked in bold.

Radionuclide	Physical Half-Life	Electron Yield per Decay	Decay Mode	γ-Photon Energy (keV)	Method of Production
^51^Cr	27.7 d	4.68	EC	320	reactor
**^67^Ga**	**78.3 h**	**7.03**	**EC**	**93,185,300**	**cyclotron**
^77^Br	57.0 d	4.96	EC	239	cyclotron
^94^Tc	4.88 h	6.42	EC	511	cyclotron
^99m^Tc	6.01 h	4.67	IT	141	cyclotron
**^111^In**	**2.82 d**	**6.05**	**EC**	**171, 245**	**cyclotron**
^114m^In	49.51d	7.74	EC	558	reactor
^115m^In	4.49 h	5.04	IT	336	reactor
^123^I	13.2 h	12.6	EC	159	cyclotron
^124^I	4.18 d	8.6	EC	511	cyclotron
**^125^I**	**59.4 d**	**21.0**	**EC**	**36**	**cyclotron**
^135^La	19.5 h	10.9	EC	485.5	cyclotron
^167^Tm	9.25 d	11.4	EC	207.8	cyclotron
**^193m^Pt**	**4.33 d**	**27.0**	**IT**	**-**	**reactor/cyclotron**
**^195m^Pt**	**4.03 d**	**37.0**	**IT**	**98.9**	**reactor/cyclotron**
**^197^Hg**	**64.1 h**	**23.2**	**EC**	**134**	**cyclotron**
**^197m^Hg**	**23.8 h**	**19.4**	**IT/EC**	**77**	**cyclotron**
^201^Tl	73.0 h	36.9	EC	68–80	cyclotron
^203^Pb	51.9 h	23.3	EC	279.2	cyclotron
